# Classifying caesarean section to understand rising rates among Palestinian refugees: results from 290,047 electronic medical records across five settings

**DOI:** 10.1186/s12884-022-05264-z

**Published:** 2022-12-13

**Authors:** Zeina Jamaluddine, Gloria Paolucci, Ghada Ballout, Hussam Al-Fudoli, Louise T. Day, Akihiro Seita, Oona M. R. Campbell

**Affiliations:** 1 Faculty of Epidemiology and Population Health, London School of Hygiene & Tropical Medicine, London, UK; 2 School of Tropical Medicine and Global Health, Nagasaki University, Nagasaki, Japan; 3 United Nations Relief and Works Agency for Palestinian Refugees in the Near East, UNRWA Headquarters, Amman, Jordan

**Keywords:** Caesarean-section, Delivery, Refugees, Obstetrics, Electronic Medical Records, Routinely Collected Health Data

## Abstract

**Background:**

Rising caesarean-section rates worldwide are driven by non-medically indicated caesarean-sections. A systematic review concluded that the ten-group classification system (Robson) is the most appropriate for assessing drivers of caesarean deliveries. Evidence on the drivers of caesarean-section rates from conflict-affected settings is scarce. This study examines caesareans-section rates among Palestinian refugees by seven-group classification, compares to WHO guidelines, and to rates in the host settings, and estimates the costs of high rates.

**Methods:**

Electronic medical records of 290,047 Palestinian refugee women using UNRWA’s (United Nations Relief and Works Agency for Palestine Refugees in the Near East) antenatal service from 2017–2020 in five settings (Jordan, Lebanon, Syria, West Bank, Gaza) were used. We modified Robson criteria to compare rates within each group with WHO guidelines. The host setting data were extracted from publicly available reports. Data on costs came from UNRWA’s accounts.

**Findings:**

Palestinian refugees in Gaza had the lowest caesarean-section rates (22%), followed by those residing in Jordan (28%), West Bank (30%), Lebanon (50%) and Syria (64%). The seven groups caesarean section classification showed women with previous caesarean-sections contributed the most to overall rates. Caesarean-section rates were substantially higher than the WHO guidelines, and excess caesarean-sections (2017–2020) were modelled to cost up to 6.8 million USD. We documented a steady increase in caesarean-section rates in all five settings for refugee and host communities; refugee rates paralleled or were below those in their host country.

**Interpretation:**

Caesarean-section rates exceed recommended guidance within most groups. The high rates in the nulliparous groups will drive future increases as they become multiparous women with a previous caesarean-section and in turn, face high caesarean rates. Our analysis helps suggest targeted and tailored strategies to reduce caesarean-section rates in priority groups (among low-risk women) organized by those aimed at national governments, and UNRWA, and those aimed at health-care providers.

**Supplementary Information:**

The online version contains supplementary material available at 10.1186/s12884-022-05264-z.

## Introduction

Medically-indicated caesarean-section is effective in reducing maternal and neonatal mortality and morbidity and stillbirths. However, the World Health Organization (WHO) suggests rates above 10% do not confer additional maternal or perinatal benefits [1, 2], while a review of ecological studies suggests that the optimal proposed caesarean-section threshold is 9–19% [3, 4]. The rising global caesarean-section rate, from 7 to 21% between 1990–2020 [5], is driven by non-medically indicated caesarean-sections [6], the latter are caesarean-sections in absence of any maternal or fetal indications such as specific pre-existent maternal health condition, low-lying placenta, placenta previa, labour dystocia, abnormal or indeterminate fetal heart rate tracing, fetal malpresentation fetal macrosomia and multiple gestations.

Non-medically indicated caesarean-sections pose unnecessary risks to mothers and children, and add financial costs. Maternal risks include haemorrhage, infections, anaesthetic and thromboembolic events, and surgical/urological complications, e.g. fistula [7, 8]. Longer term, women with caesarean delivery face increased risks of infertility, ectopic pregnancy, placental abnormalities, uterine rupture, stillbirth, preterm birth, and abdominal adhesions [9]. Children born by caesarean-section have increased risks of neonatal respiratory complications, reduced breastfeeding, and iatrogenic prematurity if gestational assessment is inaccurate. Longer-term effects on children include increased risk of mortality, asthma, and allergies, and reduced intestinal-microbiome diversity [10–13]. Hospitals in the five settings where UNRWA works charge more for caesarean sections than for vaginal births, and this pattern holds in many settings. Caesarean section also require longer hospital lengths-of-stay and recovery periods [14]. Reducing unnecessary caesarean-sections will protect women and children, improve quality of care, and reduce costs to those insuring/financing childbirth services, including women and families.

Limiting unnecessary caesarean-section requires understanding its drivers. WHO recommends the Robson classification to monitor and prioritize where caesarean-section rates should be reduced [15]. Robson categorises all women into ten mutually exclusive groups via routinely-collected clinical data on: parity, previous caesarean-section, onset of labour (spontaneous vs induced), single vs multiple pregnancy, gestational age, and fetal lie or presentation. Using ten-groups classification system (Robson) moves away from whether a caesarean-section is indicated for a specific woman, towards examining groups where rates are excessive or too low [16]. Betran and colleagues, also provide a WHO conceptual framework of drivers of high rates [17], which we used to structure our discussion.

Caesarean-section rates among Palestinian refugees have doubled from 15 to 31% between 2006–2020 [18, 19]. Refugees’ access to health services is based on their country of residence. UNRWA, the United Nations Relief and Works Agency for Palestine Refugees in the Near East, provides free primary health care services to Palestinian refugees in Jordan, Lebanon, Syria, West Bank, and Gaza, including free antenatal care services [19]. Pregnant Palestinian refugees can also access low-cost or free national public antenatal services in Jordan, West Bank, Gaza, and Syria, while in Lebanon, those not using UNRWA services must pay for private care. Except for one hospital in West Bank, UNRWA does not directly provide childbirth care– rather it used partial reimbursement co-payment schemes to accredited non-UNRWA facilities, including for childbirth.

This study examines and contextualizes caesarean-section rates among Palestinian refugees residing in the five settings where UNRWA operates, from 2017 to 2020. We examined caesareans-section rates among Palestinian refugees by using a seven groups caesarean section classification modelled on the Robson classification, compared these to WHO guidelines and to rates in the host settings, and estimated the costs of rates above the guidelines.

## Methods

We relied UNRWA electronic medical records (EMR) and cost data, and on desk reviews of annual health reports.

### The setting and UNRWA’s electronic medical records

The percentage of pregnant Palestinian refugees using UNRWA’s antenatal care services is estimated at around 35% in Jordan**,** 49% in West Bank, 73% in Gaza, 34% in Syria; it is difficult to estimate in Lebanon [19]. In Syria, many UNRWA clinics reduced services or were destroyed in the war in 2011–2014, and many Palestinian refugees left the country, making the denominator unreliable.

UNRWA’s EMR is a web-based patient-centred digital system capturing every primary healthcare clinic visit recorded by the doctor, nurse, or midwife caring for women. It includes contemporaneous modules on antenatal care, and antenatal referrals, and records pregnancy outcomes retrospectively during postnatal care, the child’s first vaccination visit, or by telephone follow-up. In 2017, the EMR system was updated to record information on the mother (place of residence, date of birth, marital status, and education level), obstetric history (parity, previous caesarean-section, previous pregnancy risk score), and more detailed current pregnancy outcomes (gestational at delivery, mode of delivery, malpresentation, number of neonates).

We extracted anonymized electronic medical records data for pregnancies ending between January 1, 2017, to December 31, 2020. Data analysis was performed in accordance with the ethics guidelines and regulations. UNRWA is the main custodian of the electronic health system and maintains the system following ethical, legal privacy and confidentiality requirements. We obtained ethics approval from UNRWA’s research review board and the London School of Health & Tropical Medicine Research Ethics Committee (LSHTM Ethics Ref: 22,801) (Date: 12 November 2020). Participant consent was not required as the study used de-identified registry based secondary data. As long as the work does not violate the rights of individuals and does not include identifiable information, UNRWA permit researchers to access EMR without obtaining prior consent from participants to pursue research for the common good of Palestinian refugees. Informed consent was waived by UNRWA’s research review board.

### Seven groups caesarean section classification

UNRWA’s EMR dataset included some variables required for the Robson classification: parity (nulliparous (nullip)/multiparous (multip)), previous caesarean-section (yes/no), number of fetuses (1/2 +), gestational age (preterm (defined as less than 37 weeks completed)/term) and fetal lie/presentation (normal/abnormal lie, malpresentation) (Appendix S1) [20], but not the specific type of malpresentation or abnormal lie (breech/transverse/oblique) or onset of labour (spontaneous/induced) or pre-labour caesarean-section. We generated a 7-groups caesarean section classification based on the Robson classification and will be refer to it as the “ 7-groups caesarean-section classification”. Therefore, we modified the Robson classification from 10 to 7 groups, collapsing group 1 (nulliparous, spontaneous labour) with group 2 (nulliparous, induced labour or caesarean-section before labour) in one group (1 + 2) and group 3 (multiparous, spontaneous labour) with group 4 (multiparous, induced labour or caesarean-section before labour) in one group (3 + 4). Hereafter these are called groups 1 + 2 (nullip, term) and groups 3 + 4 (multip, term, no prev caesarean-section). We refer to malpresentation as including both abnormal lie and malpresentation and collapsed nulliparous women in group 9 (transverse or oblique) with group 6 (breech) hereafter called groups 6/9 (nullip, malpresentation). We did the same for multiparous women group 7 (breech) with group 9 (transverse or oblique), hereafter called groups 7/9 (multip, malpresentation). Group 5 (multip, term, previous caesarean-section), group 8 (multiple pregnancy), and group 10 (preterm) were not modified. In the text the symbol “/” refers to “or” while the symbol “ + ” refers to “addition”.

Based on the Robson group-specific caesarean-section rates guidelines proposed by WHO [20], we generated a modified weighted guidelines for groups 1 + 2 (nullip, term) and groups 3 + 4 (multip, term, no previous caesarean-section) (Appendix S1).

### Desk review

Trend data from 2006 till 2020 were extracted for Palestinian refugees from UNRWA annual health reports [21], and for host settings from country national annual health reports, Palestinian Ministry of Health Annual reports (2016–2020), [22] Lebanon Ministry of Public Health (MOPH), Vital Data Observatory statistics (2015–2018) [23]. In Palestine (West Bank and Gaza), around 41% of the population are classified as refugees [24]. We also used population-based household surveys including Demographic Health Surveys (DHS) (Jordan 2007, 2012, 2017/2018) [25], Multiple Indicator Cluster Surveys (MICS) (Palestine 2010, 2014, 2019/2020, Palestinian refugees in Lebanon 2011) [26] and Pan Arab Project for Family Health (PapFam) (Syria 2009) [27]. We reviewed Health Resources Availability Monitoring System (HeRAMS) reports for facility-level data in Syria (2014 till 2018) [28].

### Cost data

Cost data from UNRWA were available to two authors (GP and AS) in their capacity as hospitalisation consultant and Director of Health Department at UNRWA.

### Analysis

We calculated frequencies and cross-tabulations to assess data quality and investigated missing data. Because the EMR reports women’s current age and parity status, we adjusted these to reflect the values when the index delivery occurred. To assess external validity, we compared our data to population surveys and national statistics.

We restricted the analysis to women who delivered a live birth at any gestation or a stillbirth at 28 completed weeks of gestation or beyond (i.e., removing early fetal deaths) [20]. Data collected in UNRWA was recorded per birth, where twins and triplets had 2 and 3 entries respectively. We collapsed twins and triplets to generate one record per delivery event (with the mother as unit of analysis) and calculated caesarean-section rates among live births, stillbirths and total births [29]. We calculated the relative size of each group among the obstetric population in each of the five settings, and the caesarean-section rates by setting, and within each group. We then calculated the absolute and relative contribution of groups to caesarean-section rates and examined the change in caesarean-section rates from 2017 to 2020. We compared caesarean-section rates within each group with WHO guidance to identify priorities for action. We then compared caesarean-section rates of refugees and host communities. All data were analysed using Stata, version 16 (StataCorp, College Station, USA).

To understand the financial impact of the caesarean section performed, we costed all deliveries as if they were performed in UNRWA contracted hospitals.

## Results

From January 1, 2017, to December 31, 2020, UNRWA EMR included data on 294,184 live births and 1,401 stillbirths which occurred in a total of 291,704 delivery events. Missing data ranged from 0.2% in Gaza to 1.5% in Syria; the final analysis data included 290,047 births (99.5%).

Most women who delivered were 20–29 years old, with Syria and Jordan having the highest prevalence of women under 20 years of age (Table 1). Gaza contributed the most deliveries (48%). Palestinian refugees residing in Jordan, Lebanon, and Syria had lower educational attainments compared to those in West Bank and Gaza (Table 1).

**Table 1:** Characteristics of Palestinian refugee women delivering in the five settings, 2017–2020

Palestinian refugees in	Jordan	Lebanon	Syria	West Bank	Gaza
Number of women delivering	72,472	15,962	15,760	47,446	140,064
Pregnancy outcome, n (%)					
Livebirth	71,979 (99.3)	15,874(99.5)	15,608(99.0)	47,275(99.6)	139,147(99.5)
Stillbirth	389 (0.5)	65(0.4)	136(0.9)	126(0.2)	667(0.5)
Unknown	104 (0.1)	23(0.1)	16(0.1)	45(0.1)	250(0.2)
Maternal age, n (%)					
< 20 years old	6219(8.6)	901(5.6)	1433(9.1)	2329(4.9)	7914(5.7)
20–24 years old	22,977(31.7)	4218(26.4)	4491(28.5)	14,662(30.9)	42,020(30.0)
25–29 years old	20,226(27.9)	5071(31.8)	4044(25.7)	15,796(33.3)	47,075(33.6)
30–34 years old	13,037(18.0)	3444(21.6)	3299(20.9)	8843(18.6)	26,819(19.1)
35–39 years old	7540(10.4)	1834(11.5)	1867(11.8)	4414(9.3)	12,674(9.0)
40–44 years old	2312(3.2)	469(2.9)	590(3.7)	1317(2.8)	3336(2.4)
> 45 years old	158(0.2)	24(0.2)	36(0.2)	82(0.2)	225(0.2)
Missing	3(0.0)	1(0.0)	0(0.0)	3(0.0)	1(0.0)
Education level, n (%)					
Illiterate/Basic	32,869(45.4)	9656(60.5)	10,169(64.5)	9576(20.2)	23,716(16.9)
Secondary	26,866(37.1)	2391(15.0)	2329(14.8)	17,593(37.1)	55,203(39.4)
Diploma	5671(7.8)	1156(7.2)	1162(7.4)	3203(6.8)	7716(5.5)
University/Higher	7066(9.7)	2759(17.3)	2100(13.3)	17,074(36.0)	53,429(38.1)
Parity, n (%)					
Nulliparous	19,386(26.7)	4828(30.2)	4923(31.2)	13,121(27.7)	33,242(23.7)
Multiparous	53,005(73.1)	11,117(69.6)	10,828(68.7)	34,289(72.3)	106,624(76.1)
Missing	81(0.1)	17(0.1)	9(0.1)	36(0.1)	198(0.1)
Previous CS, n (%)					
Nulliparous	19,386 (26.7)	4828 (30.2)	4923 (31.2)	13,121 (27.7)	33,242 (23.7)
No previous CS	39,209 (54.1)	6570 (41.2)	5095 (32.3)	25,201 (53.1)	85,127 (60.8)
Yes previous CS	13,877 (19.1)	4564 (28.6)	5742 (36.4)	9124 (19.2)	21,695 (15.5)
Number of neonates, n (%)					
Singleton	71,608(98.8)	15,715(98.5)	15,521(98.5)	46,664(98.4)	138,007(98.5)
Multiples	864(1.2)	247(1.5)	239(1.5)	782(1.6)	2057(1.5)
Foetal malpresentation, n (%)					
No	71,999(99.3)	15,897(99.6)	15,633(99.2)	46,943(98.9)	136,420(97.4)
Yes	473(0.7)	65(0.4)	127(0.8)	503(1.1)	3644(2.6)
Gestational age at delivery, n (%)					
Preterm	7929(10.9)	2023(12.7)	2400(15.2)	3852(8.1)	12,840(9.2)
Term	64,224(88.6)	13,876(86.9)	13,130(83.3)	42,949(90.5)	127,074(90.7)
Missing	319(0.4)	63(0.4)	230(1.5)	645(1.4)	150(0.1)

**Fig. 1:**
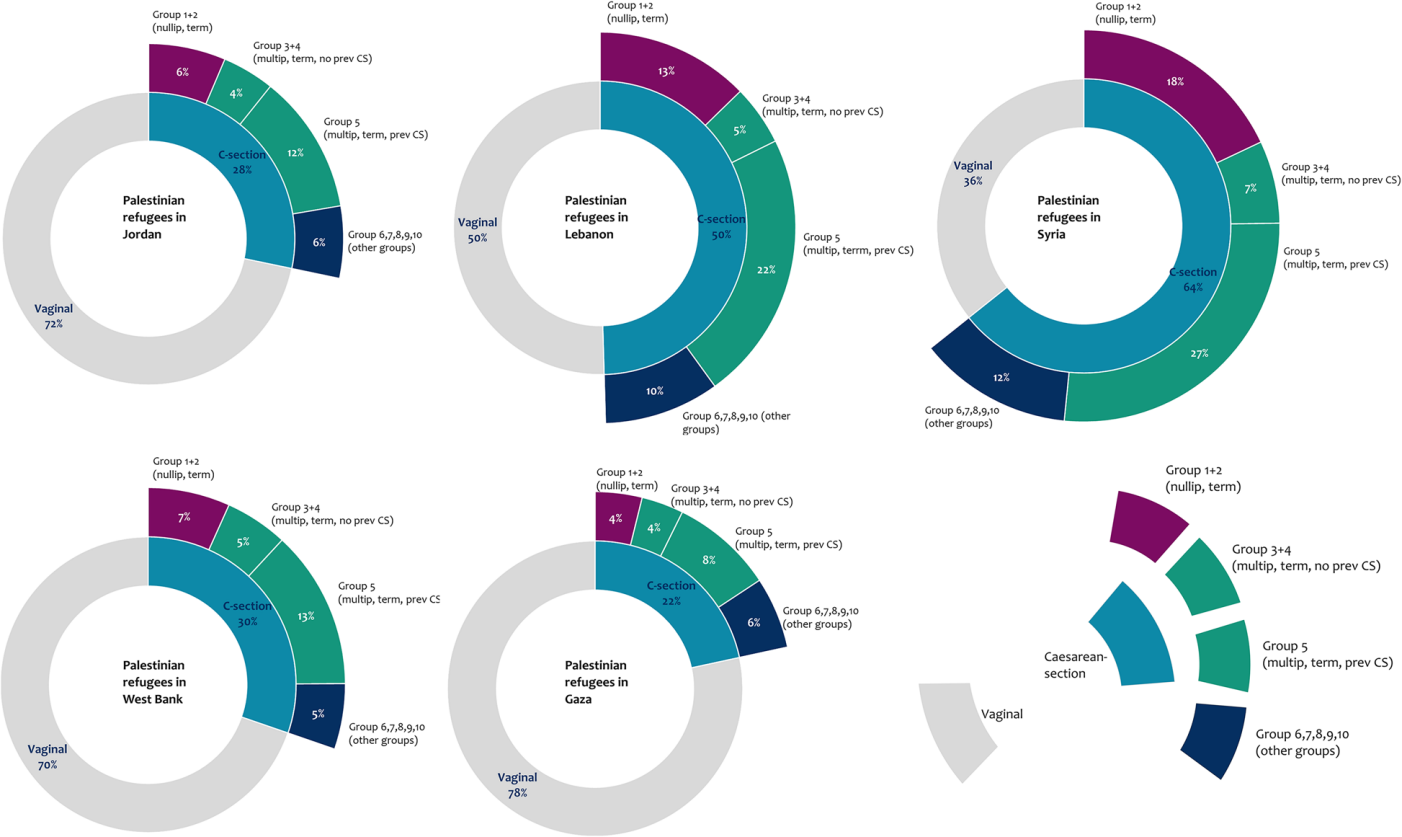
Mode of delivery in each of the five settings where Palestinian refugees reside with absolute contribution of groups to overall caesarean section rate (n = 290,047 births) (2017-2020)

Caesarean-section rates were highest for refugees in Syria (64%), followed by Lebanon (50%), West Bank (30%), and Jordan (28%) (Fig. 1). Gaza had the lowest (22%) (Fig. 1). In all five settings, caesarean-section rates among stillbirths were only slightly lower than among liveborn infants (Jordan liveborn 28% vs stillborn 25%, Lebanon liveborn 50% vs stillborn 44%, Syria liveborn 64% vs stillborn 56%, West Bank liveborn 30% vs stillborn 21%, Gaza liveborn 22% vs stillborn 19%.

**Fig. 2:**
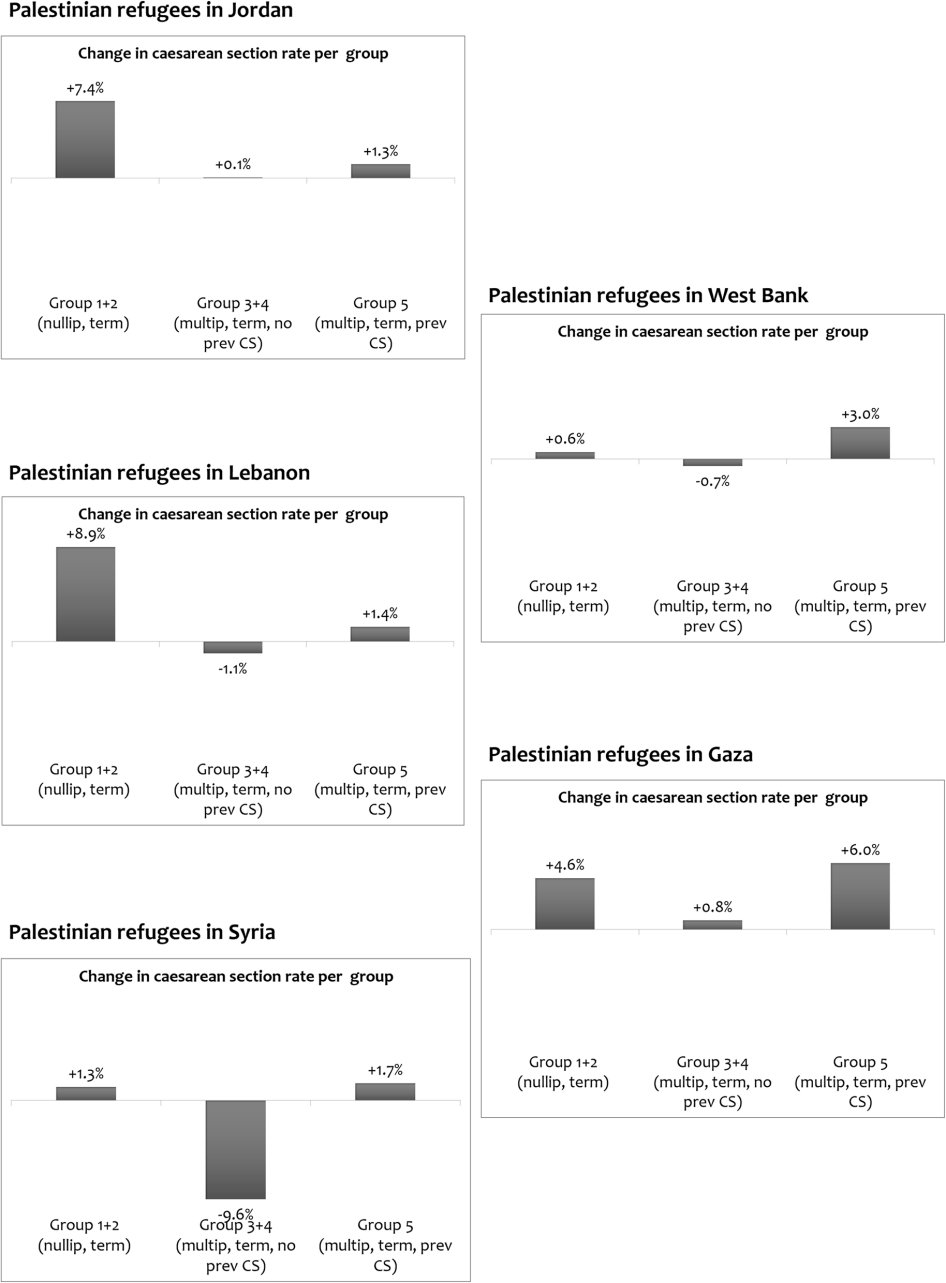
Change in caesarean section rates within caesarean section groups, in the five settings (2017–2020)

Between 2017 and 2020, caesarean-section rates increased by 1% in the West Bank 3% in Jordan, Syria, and Gaza, and 5% in Lebanon, with average annual rates of increase ranging from 0.42% to 1.94%. The largest increase in caesarean-section rates was among nulliparous women (Fig. 2).

Most pregnant women were multiparous, with a singleton pregnancy and cephalic presentation at birth. Preterm births were highest in Syria (15.2%) (Table 1). Appendix S2 includes detailed groups by setting, including total births and caesarean-sections numbers, relative group size, group caesarean-section rates, and absolute and relative contribution to the overall caesarean-section rate. The proportion of group 8 (multiple pregnancy) ranged from 1.2% in Jordan to 1.6% in West Bank (Appendix S2). The size of the multiparous groups (group 3 + 4 (multip, no prev caesarean-section) and group 5 (multip, term, prev caesarean-section)) was 64% in Jordan, 59% in Lebanon, 56% in Syria, 65% in West Bank, and 67% in Gaza, and reflected the relatively-high fertility rates in these settings (Appendix S2). Appendix S3 includes the quality of the data using 7-groups caesarean section classification.

**Fig. 3:**
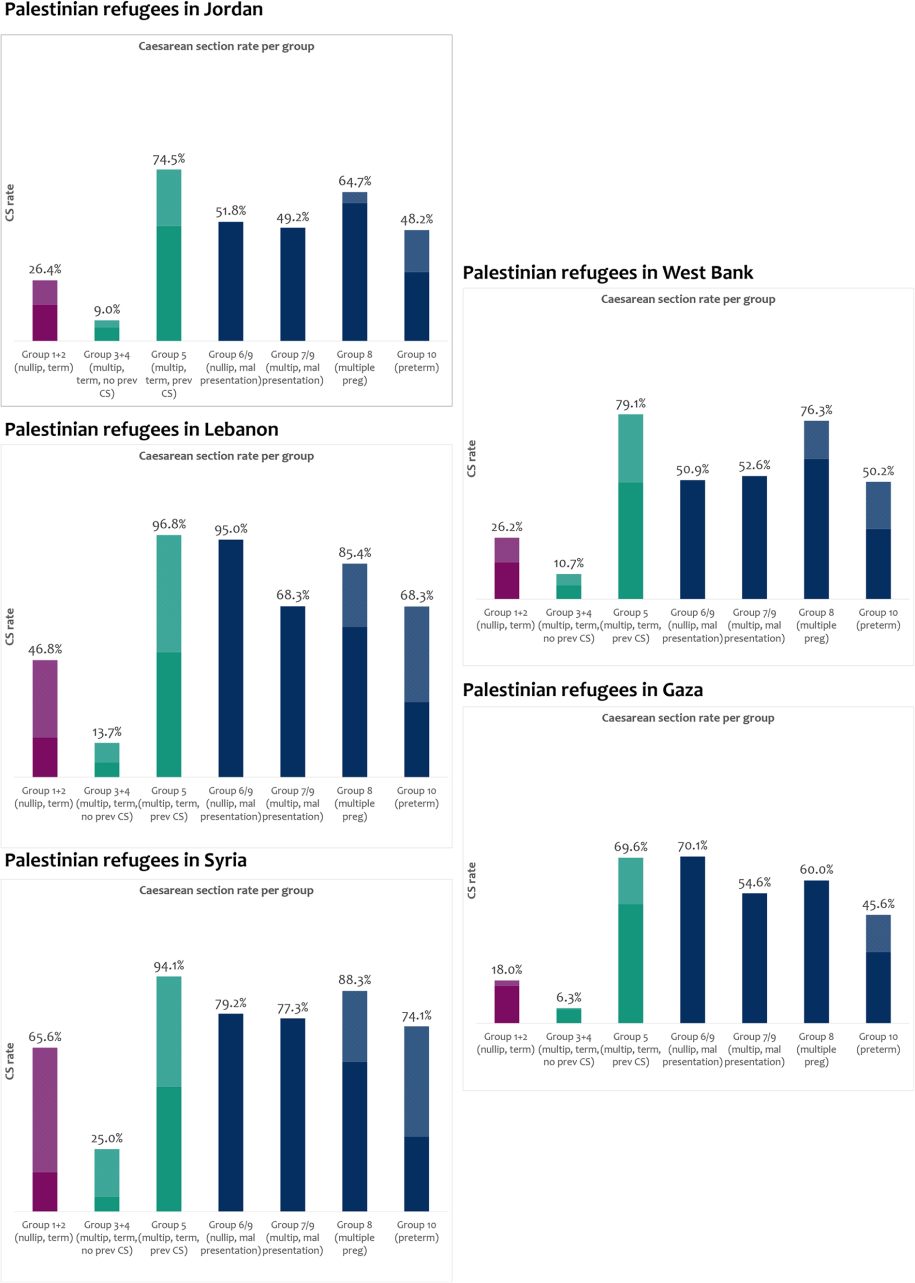
Caesarean-section group rate in five settings (*n* = 290,047 births). Shaded areas indicate percent exceeding the recommended guidelines (guidelines defines in Appendix S1)

We also examined the group contributions to the caesarean-section rate by setting. In all five settings, group 5 (multip, term, prev caesarean-section) was the highest contributor to the overall caesarean-section rate (Fig. 3). A sub-analysis on group 5 for parity one is available in Appendix S4. Caesarean-section rates in 1 + 2 groups (nullip, term), were above weighted WHO guidance of 16% in all setting (See appendix for details of weighting): Syria (65%), Lebanon (46%), Jordan (26%), West Bank (26%) and Gaza (18%) (Fig. 3).

Caesarean-section rates for group groups 3 + 4 (multip, term, no previous caesarean-section) also exceeded guidance of below 6% (guideline Appendix S1) in Jordan, Lebanon, Syria, and West Bank. Within group 5 (multip, term, previous caesarean-section), women in Lebanon and Syria had rates of 94% or over; other settings were also substantially above the 50–60% guideline (Fig. 3). All five settings had over double the recommended 30% caesarean-section rates within Robson group 10 (preterm) (Fig. 3). In groups 6/9 (nullip, malpresentation) and 7/9 (multip, malpresentation), caesarean-section rates were lower than the expected prevalence of 80–100% in most settings (Fig. 3).

**Fig. 4:**
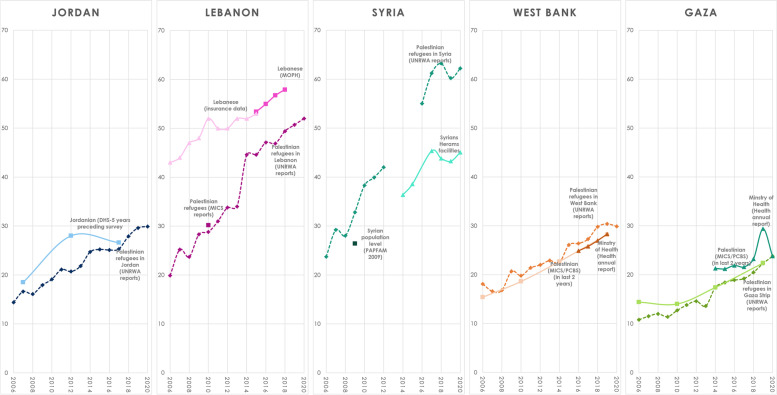
Caesarean section rates in Jordan, Lebanon, Syria among nationals and refugees and in Palestinian (West Bank and Gaza) among non-refugees and refugees from 2006 till 2020

Figure 4 compares trends in refugees to nationals (from 2006–2020) and shows increasing caesarean section rates among both Palestinian refugees and host settings. In Jordan, Lebanon, and Syria, nationals’ caesarean-section rates were higher than those of residing Palestinian refugees (Fig. 4). In the West Bank and Gaza, refugees and non-refugees had similar rates.

Table 2 shows reimbursement policies in all five settings, and results of our cost modelling of caesarean-sections that exceed the guidelines.

**Table 2:** **Reimbursement policies and cost of caesarean sections exceeding WHO guidelines.** Vaginal and caesarean-section deliveries are covered differently in the five settings as per policies shown in the table below

Average costs paid by UNRWA per patient delivery and current childbirth policy reimbursements by setting					
	Jordan	Lebanon	Syria	West Bank	Gaza
Vaginal delivery	Average USD 74 UNRWA covers 75% of the cost up to 100 JOD (equivalent to 140 USD in 2020)	Average USD 230 UNRWA covers 275,000 Lebanese Lira (Equivalent to USD 183 in 2017 and USD 15 in 2020)	Average USD 50 UNRWA covers 75% of the cost	Average USD 150 UNRWA covers 50% of the cost	Average USD 87
Caesarean section delivery	Average USD 300	Average USD 580 UNRWA covers 100% of the cost in Palestinian Red Crescent Society hospitals, 90% in Secondary hospitals, 60% in Tertiary hospitals	Average USD 100 UNRWA covers 75% of the cost	Average USD 510 UNRWA covers 75% of the cost	Average USD 430
SSN/ poor hardship	UNRWA covers 95% of the cost up to 150 JOD (equivalent to 211 USD)		UNRWA covers 95% of the cost	UNRWA covers 90% of the cost	Average 90 USD for vaginal; 445 USD for caesarean

The unit cost for deliveries that UNRWA has agreed with service providers differs according to the contract in place in the five settings. UNRWA only covers deliveries submitted within the Hospitalization Support Program and not all the deliveries recorded in its health centers. Palestinian Refugees might choose alternative solutions (governmental or private insurance scheme where available). Under the assumption that all deliveries would have cost as per UNRWA agreements, a total of USD 52.3 million would have been spent in 2017–2020: USD 20.3 million for vaginal deliveries and almost USD 32 million for caesarean-sections. USD 6.8 million of the latter amount could have been saved had guidance been adhered to (USD 1.9 million by exceeding guidance in group 1 + 2 (nullip, term), almost USD 1.4 million in group 3 + 4 (multip, term, no prev caesarean-section), USD 2 million in group 5 (multip, term, prev caesarean-section), and USD 1.5 million in group 10 (preterm))

## Discussion

We evaluated Palestinian refugee caesarean-section rates among 290,047 deliveries resulting in live birth or stillbirth from 2017–2020 in five settings. The overall caesarean rate was 28%, with considerable variability by settings. We found: (1) evidence for the need to add missing data elements in UNRWA EMR to be able to implement the ten group classification system (Robson) (2) high and increasing caesarean section rates (3) caesarean section rates within most groups exceeding guidelines, (4) a built-in momentum caused by high caesarean section rates among nulliparous women, who will likely have subsequent caesareans, (5) a powerful correlation with host country caesarean section rates, which are also increasing, (6) a considerable financial cost associated with potentially unindicated caesarean section, and (7) sub-optimal clinical management.

This study showcases the need to ensure routine EMR capture all the data necessary to fully implement the ten-groups classification system (Robson) as recommended by WHO. Our study was limited by the absence of data elements on onset of labour (spontaneous/induced) or pre-labour caesarean-section and the type of fetal abnormal lie/malpresentation type and so could not benefit from the full extent of Robson group classification, size of the group (for proper quality check) and the WHO recommendation for monitoring rates. A direct consequence of our study, is that UNRWA already added the types of presentation and onset of labour variables to its EMR, an example of collaborative research informing practice. A remaining concern however is whether this data will be captured accurately by the recently delivered women’s’ reports. This is a challenge in EMR that are collected in primary health care facilities on births which occurred elsewhere. One suggestion is that hospitals would provide all ten-group classification systems data elements in the discharge summary sheets linked to reimbursement. The EMR should also capture the number of previous cesarean-section to subdivide analysis in group 5 into group 5.1 (one previous caesarean section) and group 5.2 (the two or more previous caesarean section).

Caesarean-section rates in all five settings (22%-64%) were higher than the rates associated with improvements in maternal or neonatal outcomes or stillbirths (9–19%) [3, 4], suggesting many were not medically indicated. Over a four-year period starting in 2017, caesarean-section rates rose with an average annual increase of 0.4%-1.9%, tracking increases in host countries (average annual increases: 0.6%-1.4%).

Groups with low indication for caesarean-section, specifically the nulliparous women group (1 + 2) and the multiparous women without a previous caesarean-section group (3 + 4), had rates that were higher than WHO guidelines.

The rapid rise in caesarean-section rates over time stems partly from the increase in rates of first caesarean-section among the nulliparous group (group 1 + 2), combined with high fertility, whereby these women go on to have multiple subsequent births. Palestinian refugees have relatively high total fertility rates, around 3.6 births per women in West Bank and Gaza in 2020, 3.3 in Jordan in 2010, 2.7 in Lebanon in 2017, 2.5 in Syria in 2011 [30]. Reflecting this high fertility, the multiparous obstetric population in our study is 69%-76%, a relatively high percentage compared to other countries, and higher than WHO expectations of approximately 58%-65% [31]. High fertility rates, combined with increases in unnecessary caesarean birth among nulliparous women, have a built-in momentum and lead to future increases in caesarean births as these women subsequently enter group 5 (multip, term, prev caesarean-section) who in turn have high caesarean section rates. This group contributed the most to the high caesarean-section rates (8–27%).

The findings from previous studies in Lebanon and Gaza using Robson to classify caesarean-section, are comparable to our results [32, 33]. However, the former analyses were restricted to Lebanese women using a tertiary hospital in Lebanon and Palestinian women in three hospitals in Gaza, while our study is population based [32, 33].

In the Palestinian refugee context, there is an urgent need to develop strategies targeting low-risk women to optimise vaginal delivery and limit an even more rapid rise in caesarean-section rates, as well as a need to improve quality of care. We use the framework of Betran and colleagues, which identifies interventions and strategies at different levels to discuss potential approaches: namely those linked to national governments and health-care organizations and those linked to health professionals [17].

### Drivers and interventions linked to national systems and healthcare organisations

The high rates and increasing trends in caesarean-section seen in the refugee population do not occur in a vacuum – rather they reflect rates and trends in the national host populations. In West Bank and Gaza, rates among Palestinian refugees and non-refugees are comparable, suggesting there are not major differences in these populations. In Jordan, Lebanon and Syria, the lower rates seen among refugees compared to nationals probably reflect the lower socio-economic status and greater marginalization of refugees, or possibly the rates in the hospitals which UNRWA contracts out.

The high and increasing national trends in all five settings also reflect the structure of national health systems. There are low and declining percentages of births attended by midwives. For example, 23.8% of deliveries in Jordan in the 2012 DHS were with midwives, declining to 10.6% in the 2017–18 DHS. The most recent survey data suggest births attended by doctors (largely obstetricians) are 89.1% in Jordan (2017–18), 86.7% in Lebanon (2011), around 80% in Syria (2009 pre-conflict), 70.8% in West Bank (2019–20) and 87.5% in Gaza (2019–20) [27, 34–36]. The high rates we observed among refugees and Syrians in Syria might stem from the conflict, which reduced access and availability of obstetricians [37], and shifted to delivery by general surgeons, who only conduct caesarean deliveries.

Health systems in the five settings also have substantial and growing doctor-led private sectors (33.4% Jordan (2017–18), 29.1% Lebanon (2011), 46.3% Syria (2009), 46.3% West Bank (2019–20) and 15.7% Gaza (2019–20)) [27, 34–36].Private providers earn more from caesarean deliveries, and find them more manageable to schedule because women expect to be delivered by “their” doctor [38].

Costs associated with unindicated caesarean sections are substantial, particularly in the context of large and ever-growing numbers of refugees and donor fatigue. Unnecessary costs also burden national governments and women and their families, irrespective of refugee/national status.

Our results highlight the need to intervene with a unified national effort/policy to decrease caesarean-section for both refugees and women in the national host settings, particularly where donors do not fund their own services, but rather contract them out to national providers. Refugee agencies, namely UNRWA and UNHCR, may also have opportunities to use their financial clout in contracting and negotiating with hospitals. Strategies proposed in the literature at national level include removing perverse financial incentives or changing the percentage coverage of caesarean-sections to avoid privileging these over vaginal births [17].

### Interventions targeting health professionals and facility managers

In addition to the excessive rates within most of the categories which were discussed above, we also found evidence of likely suboptimal clinical decisions by health-care providers, including high rates of caesarean-section among stillbirths and low rates of vaginal birth after caesarean (VBAC).

There were high, and increasing, caesarean-section rates among stillbirths (19%-56%). This is higher than rates observed in some high income countries (United State of America (15% in 2014)) and low middle income countries (7% in 2015) [39, 40]. Timely emergency caesarean-section is indicated when fetal distress is diagnosed. However unless there is a maternal medical indication, vaginal birth is the recommended mode of delivery when the baby is known to have died in utero [41]. Women’s preferences should also be taken into account with a discussion of the risk–benefit balance for subsequent pregnancies after caesarean-section and reassurance of support during vaginal birth including adequate analgesia.

Trial of labour after caesarean (TOLAC) and VBAC are uncommon among Palestinian refugees and in the host countries, where “once a caesarean always a caesarean”, is embraced. In our study, 3.2%-30.4% of multiparous women with previous caesarean section had a vaginal birth, and VBAC was particularly low in Lebanon (3.2%) and Syria (5.9%). Some of the benefits of VBAC documented included lower rates of maternal morbidity and mortality and shorter recovery compared to repeated caesarean-sections [42]. Repeated caesarean-sections in the context of high parity pose complications including abnormal placental adherence, bowel, and bladder injury and may require emergency hysterectomy [43], in the index pregnancy [44], and have serious implications for future pregnancies [43].

Both aspects of suboptimal care require support to health providers to improve clinical management. Currently recommended interventions include: educational training, exploring improving adherence to evidence-based practices, caesarean-section decision second opinions policies, clinical audit, and feedback [17]. In terms of education, addressing safety concerns, misinformation, convenience, and peer group norms among clinicians in the decision-making related to vaginal delivery of stillbirths and to TOLAC and VBAC could help in reducing caesarean-section rates [17]. In different countries, including Lebanon, a policy for a mandatory second opinion along with audit system and feedback has been implemented to reduce unnecessary caesarean-section, including by UNHCR which supports Syrian refugees in Lebanon. This second opinion approach by UNCHR led to a flattening in the rates of caesarean-section increase [45]. In Lebanon, Médecins Sans Frontières provides childbirth services directly, attempting to model more evidence-based, less interventionist childbirth practices via a midwifery-led birthing centre in Lebanon, adjacent to the largest public hospital in Beirut.

The literature also suggests provider feedback, showcasing caesarean-section rates using Robson groups in a standardised, and action-oriented manner helps health professional staff see their institutional rates and could also be used as an audit tool [17]. Facility managers and accrediting bodies can also be approached with such data, as there is evidence that facilities may be encouraging high caesarean-section rates [46].

The conceptual framework we used assessed drivers and interventions at the community/family/woman level and gave options to target women with information to address concerns about safety, misinformation, or mode of delivery choices. Our study did not gather evidence from women or communities, but there is no evidence that substantial proportions of refugee women are requesting unnecessary caesarean-sections; moreover, women are at a relative disadvantage in the power dynamics of decision making vis-à-vis health providers [47]. A recommendation for future research would be to collect data on this dimension of caesarean-section.

Despite most Robson categories having excessive rates, we were surprised to find caesarean-section rates in the malpresentation groups were lower than the Robson guideline. This might be due to misclassification of malpresentation which was captured at the last antenatal visit not labour, or because of our merging of transverse lie with breech.

## Conclusion

The most successful caesarean-section reduction strategies are multi-faceted. We show that Palestinian refugees caesarean-section rates in five settings were higher than the recommended levels in the group where caesarean-section is unindicated. The analysis indicates the need to collect further information on malpresentation and induction to conduct the 10 groups caesarean classifications (Robson). Our analysis suggests areas where targeted and tailored strategies can be applied to reduce caesarean-section rates in priority groups (among low-risk women) organized by those aimed at national governments, and UNRWA, and those aimed at health-care providers.

## Authors’ contributors

OC and AS conceived the study. GP, GB, HA extracted the data. ZJ and HA cleaned the data. ZJ and GP analysed the data, with guidance from OC. ZJ, GP, OC wrote the first draft of the manuscript. LTD supported in interpreting the findings and writing the analysis. All authors read and approved the final version.

## Supplementary Information

**Additional file 1.**

## Data Availability

Data for this study, including individual participant data and variables in the dataset, may be shared, depending on specific requests. Refugees are in a vulnerable position and their information is highly sensitive. UNRWA, as the main collector of personal data of Palestinian refugees, has a system in place to ensure respect for data protection principles. De-identified participant data, and the statistical analysis plan, may be shared for specific analyses within an agreed-upon collaboration under the terms of a signed access agreement with the two joint senior authors (AS, OC). The request for data will be reviewed by an independent research review board at UNRWA.
