# Supplementary material for: Classifying caesarean section to understand rising rates among Palestinian refugees: results from 290,047 electronic medical records across five settings
Source: BMC Pregnancy Childbirth. 2022 Dec 13;22:935. doi: 10.1186/s12884-022-05264-z (PMC9746094; doi:10.1186/s12884-022-05264-z)
Supplement: Supplementary file 1 — Additional file 1. [file 12884_2022_5264_MOESM1_ESM.docx]

**Supplementary Material content**

[**Appendix S1- Caesarean section classification** 2](#_Toc118747734)

[**Appendix S2-Detailed caesarean section classification in all five settings, 2017-2020** 3](#_Toc118747735)

[**Number of women in group** 3](#_Toc118747736)

[**Appendix S3- Assessment of data quality using the Group Classification Report Table.** 8](#_Toc118747737)

[**Appendix S4- Group5 sub-analysis.** 9](#_Toc118747738)

# **Appendix S1- Caesarean section classification**

| **group** | **Description of obstetric populations** | Parity | Previous caesarean-section | Number of neonates | Foetal presentation | Gestational weeks | Guideline |
| --- | --- | --- | --- | --- | --- | --- | --- |
| Group 1+2  (null, term) | Nulliparous, single cephalic, >37 weeks, in spontaneous or induced labour or caesarean-section before labour | 0 | No | 1 | Cephalic | >37 | 13·3%-18·3%  Midpoint  15.8% ^a^ |
| Group 3+4  (multip, term, no prev CS) | Multiparous (no prev. caesarean-section), single cephalic, >37 weeks, in spontaneous or induced labour or caesarean-section before labour | >1 | No | 1 | Cephalic | >37 | 6.0% ^b^ |
| Group 5  (multip, term, prev CS) | Previous caesarean-section, single cephalic, >37 weeks | >1 | Yes | 1 | Cephalic | >37 | 50-60% ^c^ |
| Group 6/9  (nulip, mal-presentation) | All nulliparous malpresentation | 0 | No | 1 | Malpresentation | >37 or <37 | 80-100% ^d^ |
| Group 7/9  (multip, mal-presentation | All multiparous malpresentation (including with prev. caesarean-section) | >1 | No or Yes | 1 | Malpresentation | >37 or <37 | 80-100% ^d^ |
| Group 8  (multiple pregnancy) | All multiple pregnancies (including with prev. caesarean-section) | 0 or >1 | No or Yes | >2 | Any | >37 or <37 | 60% ^c^ |
| Group 10  (preterm) | All single cephalic, <36 weeks (including with prev. caesarean-section) | 0 or >1 | No or Yes | 1 | Cephalic | <37 | 30% ^c^ |

^a^ The ratio of Group group 1 and Group group 2 obstetric size is 2:1.

- WHO guidelines ^20^ Group group 1: CS rates under 10%
- WHO guidelines ^20^ Group group 2: CS rates around 20-35%

Combined rates based on ratio 13·3%-18·3%. Midpoint 15·8%.

^b^ The ratio of Group group 3 and Group group 4 obstetric size is 3:1

- WHO guidelines ^20^ Group group 3: CS rates under 3%
- WHO guidelines ^20^ Group group 4: CS rates under 15%

Combined the rates based on ratio 6%

^c^ Based on WHO guidelines. ^20^

^d^ Based on levels in high income countries. ^21^

# **Appendix S2-Detailed caesarean section classification in all five settings, 2017-2020**

Palestinian refugees in Jordan

| **Group group** | **Description of obstetric populations** | **TOTAL 2017-2020** | | | | | |
| --- | --- | --- | --- | --- | --- | --- | --- |
|  |  | **Number of caesarean deliveries in group** | **Number of women in group** | **Relative size of group** | **CS rate in each group** | **Absolute contribution of each group to the overall CS rate** | **Relative contribution of each group to the overall CS rate** |
|  |  | **n** | **N** | **(%)** | **(%)** | **(%)** | **(%)** |
| Group 1+2  (nullip, term) | Nulliparous, single cephalic, >=37 weeks, in spontaneous labour or, induced or CS before labour | 4,618 | 17,491 | 24·3% | 26·4% | 6·4% | 22·7% |
| Group 3+4  (multip, term, no prev CS) | Multiparous (excluding prev· CS), single cephalic, >=37 weeks, in spontaneous labour or induced or CS labour | 3,129 | 34,816 | 48·3% | 9·0% | 4·3% | 15·3% |
| Group 5  (multip, term, prev CS) | Previous CS, single cephalic, >=37 weeks | 8,333 | 11,182 | 15·5% | 74·5% | 11·6% | 40·9% |
| Group 6+9  (nullip, malpresentation) | All nulliparous malpresentation | 58 | 112 | 0·2% | 51·8% | 0·1% | 0·3% |
| Group 7+9  (multip, malpresentation | All multiparous malpresentation (including prev· CS) | 155 | 315 | 0·4% | 49·2% | 0·2% | 0·8% |
| Group 8  (multiple preg) | All multiple pregnancies (including prev· CS) | 554 | 856 | 1·2% | 64·7% | 0·8% | 2·7% |
| Group 10  (preterm) | All single cephalic, <=36 weeks (including prev· CS) | 3,541 | 7,343 | 10·2% | 48·2% | 4·9% | 17·4% |
|  | **Total** | **20,388** | **72,115** | **100.0%** | **28.3%** | **28.3%** | **100.0%** |

Palestinian refugees in Lebanon

| **Group group** | **Description of obstetric populations** | **TOTAL 2017-2020** | | | | | |
| --- | --- | --- | --- | --- | --- | --- | --- |
|  |  | **Number of caesarean deliveries in group** | **Number of women in group** | **Relative size of group** | **CS rate in each group** | **Absolute contribution of each group to the overall CS rate** | **Relative contribution of each group to the overall CS rate** |
|  |  | **n** | **N** | **(%)** | **(%)** | **(%)** | **(%)** |
| Group 1+2  (nullip, term) | Nulliparous, single cephalic, >=37 weeks, in spontaneous labour or, induced or CS before labour | 2,020 | 4,320 | 27·2% | 46·8% | 12·7% | 25·7% |
| Group 3+4  (multip, term , no prev CS) | Multiparous (excluding prev· CS), single cephalic, >=37 weeks, in spontaneous labour or induced or CS labour | 790 | 5,748 | 36·2% | 13·7% | 5·0% | 10·0% |
| Group 5  (multip, term, prev CS) | Previous CS, single cephalic, >=37 weeks | 3,548 | 3,666 | 23·1% | 96·8% | 22·3% | 45·1% |
| Group 6/9  (nullip, malpresentation) | All nulliparous malpresentation | 19 | 20 | 0·1% | 95·0% | 0·1% | 0·2% |
| Group 7/9  (multip, malpresentation | All multiparous malpresentation (including prev· CS) | 28 | 41 | 0·3% | 68·3% | 0·2% | 0·4% |
| Group 8  (multiple preg) | All multiple pregnancies (including prev· CS) | 210 | 246 | 1·5% | 85·4% | 1·3% | 2·7% |
| Group 10  (preterm) | All single cephalic, <=36 weeks (including prev· CS) | 1,260 | 1,846 | 11·6% | 68·3% | 7·9% | 16·0% |
|  | **Total** | **7,875** | **15,887** | **100.0%** | **49.6%** | **49.6%** | **100.0%** |

Palestinian refugees in Syria

| **Group group** | **Description of obstetric populations** | **TOTAL 2017-2020** | | | | | |
| --- | --- | --- | --- | --- | --- | --- | --- |
|  |  | **Number of caesarean deliveries in group** | **Number of women in group** | **Relative size of group** | **CS rate in each group** | **Absolute contribution of each group to the overall CS rate** | **Relative contribution of each group to the overall CS rate** |
|  |  | **n** | **N** | **(%)** | **(%)** | **(%)** | **(%)** |
| Group 1+2  (nullip, term) | Nulliparous, single cephalic, >=37 weeks, in spontaneous labour or, induced or CS before labour | 2,789 | 4,254 | 27·4% | 65·6% | 18·0% | 27·9% |
| Group 3+4  (multip, term , no prev CS) | Multiparous (excluding prev· CS), single cephalic, >=37 weeks, in spontaneous labour or induced or CS labour | 1,058 | 4,240 | 27·3% | 25·0% | 6·8% | 10·6% |
| Group 5  (multip, term, prev CS) | Previous CS, single cephalic, >=37 weeks | 4,163 | 4,423 | 28·5% | 94·1% | 26·8% | 41·7% |
| Group 6/9  (nullip, malpresentation) | All nulliparous malpresentation | 19 | 24 | 0·2% | 79·2% | 0·1% | 0·2% |
| Group 7/9  (multip, malpresentation | All multiparous malpresentation (including prev· CS) | 75 | 97 | 0·6% | 77·3% | 0·5% | 0·8% |
| Group 8  (multiple preg) | All multiple pregnancies (including prev· CS) | 204 | 231 | 1·5% | 88·3% | 1·3% | 2·0% |
| Group 10  (preterm) | All single cephalic, <=36 weeks (including prev· CS) | 1,672 | 2,256 | 14·5% | 74·1% | 10·8% | 16·8% |
|  | **Total** | **9,980** | **15,525** | **100.0%** | **64.3%** | **64.3%** | **100.0%** |

Palestinian refugees in West Bank

| **Group group** | **Description of obstetric populations** | **TOTAL 2017-2020** | | | | | |
| --- | --- | --- | --- | --- | --- | --- | --- |
|  |  | **Number of caesarean deliveries in group** | **Number of women in group** | **Relative size of group** | **CS rate in each group** | **Absolute contribution of each group to the overall CS rate** | **Relative contribution of each group to the overall CS rate** |
|  |  | **n** | **N** | **(%)** | **(%)** | **(%)** | **(%)** |
| Group 1+2  (nullip, term) | Nulliparous, single cephalic, >=37 weeks, in spontaneous labour or, induced or CS before labour | 3,149 | 12,002 | 25·7% | 26·2% | 6·7% | 22·2% |
| Group 3+4  (multip, term , no prev CS) | Multiparous (excluding prev· CS), single cephalic, >=37 weeks, in spontaneous labour or induced or CS labour | 2,412 | 22,485 | 48·1% | 10·7% | 5·2% | 17·0% |
| Group 5  (multip, term, prev CS) | Previous CS, single cephalic, >=37 weeks | 6,092 | 7,704 | 16·5% | 79·1% | 13·0% | 43·0% |
| Group 6/9  (nullip, malpresentation) | All nulliparous malpresentation | 56 | 110 | 0·2% | 50·9% | 0·1% | 0·4% |
| Group 7/9  (multip, malpresentation | All multiparous malpresentation (including prev· CS) | 180 | 342 | 0·7% | 52·6% | 0·4% | 1·3% |
| Group 8  (multiple preg) | All multiple pregnancies (including prev·CS) | 589 | 772 | 1·6% | 76·3% | 1·3% | 4·2% |
| Group 10  (preterm) | All single cephalic, <=36 weeks (including prev·CS) | 1,694 | 3,373 | 7·2% | 50·2% | 3·6% | 12·0% |
|  | **Total** | **14,172** | **46,788** | **100.0%** | **30.3%** | **30.3%** | **100.0%** |

Palestinian refugees in Gaza

| **Group group** | **Description of obstetric populations** | **TOTAL 2017-2020** | | | | | |
| --- | --- | --- | --- | --- | --- | --- | --- |
|  |  | **Number of caesarean deliveries in group** | **Number of women in group** | **Relative size of group** | **CS rate in each group** | **Absolute contribution of each group to the overall CS rate** | **Relative contribution of each group to the overall CS rate** |
|  |  | **n** | **N** | **(%)** | **(%)** | **(%)** | **(%)** |
| Group 1+2  (nullip, term) | Nulliparous, single cephalic, >=37 weeks, in spontaneous labour or, induced or CS before labour | 5,385 | 29,925 | 21·4% | 18·0% | 3·9% | 17·8% |
| Group 3+4  (multip, term , no prev CS) | Multiparous (excluding prev· CS), single cephalic, >=37 weeks, in spontaneous labour or induced or CS labour | 4,831 | 76,366 | 54·7% | 6·3% | 3·5% | 16·0% |
| Group 5  (multip, term, prev CS) | Previous CS, single cephalic, >=37 weeks | 11,761 | 16,894 | 12·1% | 69·6% | 8·4% | 38·9% |
| Group 6/9  (nullip, malpresentation) | All nulliparous malpresentation | 446 | 636 | 0·5% | 70·1% | 0·3% | 1·5% |
| Group 7/9  (multip, malpresentation | All multiparous malpresentation (including prev·CS) | 1,394 | 2,552 | 1·8% | 54·6% | 1·0% | 4·6% |
| Group 8  (multiple preg) | All multiple pregnancies (including prev· CS) | 1,226 | 2,043 | 1·5% | 60·0% | 0·9% | 4·1% |
| Group 10  (preterm) | All single cephalic, <=36 weeks (including prev· CS) | 5,159 | 11,316 | 8·1% | 45·6% | 3·7% | 17·1% |
|  | **Total** | **30,202** | **139,732** | **100.0%** | **21.6%** | **21.6%** | **100.0%** |

| **Step** | **Group guideline** | **Jordan** | **Lebanon** | **Syria** | **West Bank** | **Gaza** | **Interpretation** |
| --- | --- | --- | --- | --- | --- | --- | --- |
| Size of group (1+2)  Nulliparous | 35-42% | 24·3 | 27·2 | 27·4 | 25·7 | 21·4 | LOW: Nulliparous women less than range so fertility is high |
| Size of group Multiparous no previous CS (3+4) | Approx·30% | 48·3 | 36·2 | 27·3 | 48·1 | 54·7 | HIGH: Multiparous women > range so fertility is high  (see size of group 5 as well) |
| Size of group Multiparous with previous CS (5) | Roughly 1/2 total CS rate· <10% in low CS settings | 15·5 | 23·1 | 28·5 | 16·5 | 12·1 | FINE: Trend is increasing· |
| Size of group Malpresentation (6+7+9) | ~3-4% | 0·6 | 0·4 | 0·8 | 0·9 | 3·3 | LOW: Possible poor data quality linked to the source of the data relying on women’s self report· |
| Size of group Multiple pregnancy (8) | 1·5-2% | 1·2 | 1·5 | 1·5 | 1·6 | 1·5 | FINE |
| Size group preterm (10) | less than 5% | 10·2 | 11·6 | 14·5 | 7·2 | 8·1 | HIGH: High levels of preterm but (mis classify and affect 6&7) |
| Ratio group 6:7 | Usually 2:1; breeches more in nulliparous than multiparous | 0·5 | 0·33 | 0·33 | 0·28 | 0·25 | LOW: Data errors· But follows groups 1&2 and 3&4 ratio |

# **Appendix S3- Assessment of data quality using the Group Classification Report Table.**

# **Appendix S4- Group5 sub-analysis.**

Group5 can be further sub-analysed as group 5.1 (having one previous CS) group 5.2 (having 2 or more previous CS)

In the dataset it is not possible to know how many previous CS women had, only 1, or 2 or more (group 5.2 cannot be generated),

We did a sub-analysis based on women during ANC with previous CS who were Para (as their only previous delivery had been by c-section (group 5.1)).

We found in all 5 settings, CS are higher in group 5.1 (one previous CS for those with Para 1) than in parity 2 plus with one or more CS

| **Palestinian refugees in Jordan** | | **Palestinian refugees in Lebanon** | | **Palestinian refugees in Syria** | | **Palestinian refugees in West Bank** | | **Palestinian refugees in Gaza** | |
| --- | --- | --- | --- | --- | --- | --- | --- | --- | --- |
| Parity is 1  Group 5.1 | Parity more than 1 | Parity is 1  Group 5.1 | Parity more than 1 | Parity is 1  Group 5.1 | Parity more than 1 | Parity is 1  Group 5.1 | Parity more than 1 | Parity is 1  Group 5.1 | Parity more than 1 |
| 80.8% | 71.9% | 97.9% | 95.8% | 96.2% | 92.2% | 82.8% | 77.3% | 75.6% | 67.5% |
